# STNet: A Time-Frequency Analysis-Based Intrusion Detection Network for Distributed Optical Fiber Acoustic Sensing Systems

**DOI:** 10.3390/s24051570

**Published:** 2024-02-29

**Authors:** Yiming Zeng, Jianwei Zhang, Yuzhong Zhong, Lin Deng, Maoning Wang

**Affiliations:** 1 College of Computer Science, Sichuan University, Chengdu 610065, China; zengyiming@stu.scu.edu.cn; 2 National Key Laboratory of Fundamental Science on Synthetic Vision, Sichuan University, Chengdu 610064, China; zhangjianwei@scu.edu.cn (J.Z.); denglin@stu.scu.edu.cn (L.D.); 3 College of Electrical Engineering, Sichuan University, Chengdu 610065, China; zyzc122@scu.edu.cn

**Keywords:** deep learning, distributed optical fiber acoustic sensing (DAS), intrusion detection, Stockwell transform, time-frequency analysis

## Abstract

Distributed optical fiber acoustic sensing (DAS) is promising for long-distance intrusion-anomaly detection tasks. However, realistic settings suffer from high-intensity interference noise, compromising the detection performance of DAS systems. To address this issue, we propose STNet, an intrusion detection network based on the Stockwell transform (S-transform), for DAS systems, considering the advantages of the S-transform in terms of noise resistance and ability to detect disturbances. Specifically, the signal detected by a DAS system is divided into space–time data matrices using a sliding window. Subsequently, the S-transform extracts the time-frequency features channel by channel. The extracted features are combined into a multi-channel time-frequency feature matrix and presented to STNet. Finally, a non-maximum suppression algorithm (NMS), suitable for locating intrusions, is used for the post-processing of the detection results. To evaluate the effectiveness of the proposed method, experiments were conducted using a realistic high-speed railway environment with high-intensity noise. The experimental results validated the satisfactory performance of the proposed method. Thus, the proposed method offers an effective solution for achieving high intrusion detection rates and low false alarm rates in complex environments.

## 1. Introduction

Distributed optical fiber acoustic sensing (DAS) utilizes optical fibers to monitor ambient vibrations. The vibration data collected by the monitoring DAS system reflect the spatiotemporal strain of the corresponding optical fibers resulting from the vibration source. Owing to their small size, long detection distance, strong positioning accuracy, good concealment, and anti-interference, DAS systems have been widely used in seismic wave detection [1,2], power cables [3], vehicle tracking [4,5], traffic flow [6,7,8], railway intrusion monitoring [9,10,11], pipelines [12,13,14], marine structure monitoring [15], and other fields.

Many studies have addressed the detection and classification of DAS vibration events using machine-learning methods. A common thread in these studies is the detection of vibration events using feature-extraction techniques (such as the Fourier transform [2], short-time Fourier transform (STFT) [16], wavelet packet decomposition (WPD) [13], and principal component analysis (PCA) [4]), along with classifiers (for example, support vector machines (SVMs) [17], hidden Markov models (HMMs) [13], Gaussian mixture models (GMMs) [16,18], and XGBoost [9]). Because the vibration signals captured by a typical DAS system reflect the essential characteristics of the corresponding vibration source, these methods are feasible; consequently, satisfactory results have been achieved. However, most machine-learning algorithms are designed to work on relatively simple data that consist of only a few features. In long-distance detection tasks, the amount of signal data captured by a typical DAS system is very large; in addition, the collected data are often corrupted by noise, which may significantly affect the performance of the existing machine-learning methods.

With the advent of deep-learning methods, many researchers have applied convolutional neural networks (CNNs) to the detection and classification of DAS vibration events. In general, these approaches automatically extract deep features from DAS vibration data and fit optimal classification algorithms without manual settings. In the method proposed by Xu [19], the DAS signal is first denoised using spectral subtraction, and the STFT is then used for converting the signal into a time-frequency spectrum matrix, which is presented to a CNN. For this method, the recognition rates for four types of vibration events exceeded 90%. In the method proposed by Shi [20], the DAS signal was converted into a mel-scale frequency cepstral coefficients matrix (MFCC); this matrix was then combined with the original space–time data matrix for presentation to AlexNet and Inception-v3; these networks reached classification accuracies of 99.55% and 97.95%, respectively. Tian et al. [21] introduced the attention mechanism and combined it with a bidirectional long short-term memory model (ATCN-BiLSTM) to classify the DAS vibration events; the average classification accuracy was 99.6%.

In those previous studies [20,21,22], data were collected in idealistic laboratory environments or at closed sites. Consequently, the characteristics of the acquired DAS signals were clearly detectable, and the background was nearly without any interference noise. However, realistic settings feature different types of interference noise; this problem has not been sufficiently addressed in previous studies. Li et al. [10] developed a model that combined a CNN and an LSTM and applied them to a dataset collected in a real high-speed railway environment. The CNN analyzed the DAS signals and extracted their spatial characteristics, whereas the LSTM extracted the temporal characteristics of the signals. An intrusion threat-detection rate of 85.6% with a false alarm rate (FAR) of 8.0% was achieved. However, the recognition performance of the original system that used only the space–time information was not ideal and could be easily affected by high-intensity interference noise.

Therefore, accurately identifying and locating intrusion events and reducing the FAR when operating in realistic environments where noise is generated at high frequencies is a major technical challenge facing DAS systems. This paper proposes a general time-frequency analysis-based intrusion detection framework for DAS systems. First, signals that are captured by the DAS system are divided into scanning frames. Then, a window slides across the scanning frames to generate overlapping space–time windows, which are subsequently transformed into multi-channel time-frequency feature matrices using a time-frequency analysis method. Finally, the feature matrices are presented to the proposed STNet. To ensure satisfactory performance, the positions at which intrusions occur have to be located with maximal possible precision. Therefore, we used a non-maximum suppression algorithm (NMS) to post-process the detection results. Experiments were conducted on the data that were collected at a high-speed railway, which is a typical complex environment in which intrusion-related signals may be mixed with vibration signals generated by passing trains or heavy trucks, and with interference noise owing to surrounding objects. Three types of intrusion actions were simulated: (1) climbing a fence, (2) shaking a barbed wire, (3) and breaking a wall. The experimental results show that the proposed method effectively detected intrusion events and avoided false alarms owing to the interference noise, performing better than other existing methods and models.

The remainder of the paper is organized as follows: Section 2 introduces the intrusion-detection framework based on the time-frequency analysis method, whereas Section 3 presents the experimental process and the method for computing the performance-evaluation metric. Section 4 presents the analysis of the experimental results and compares the proposed method with other methods and models. Section 5 presents the conclusions and future prospects.

## 2. Principle of Intrusion Detection

### 2.1. Intrusion Detection System

**Figure 1:**
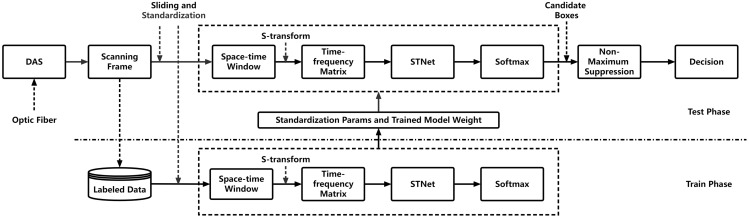
Structure of the proposed framework for detecting intrusions.

The proposed intrusion detection system for DAS vibration signals is shown schematically in Figure 1; the figure shows the training and testing phases. The vibration signals captured by the DAS system are initially segmented into scanning frames based on a manually determined scanning period and then divided into training and testing sets.

During the training phase, z-score standardization is applied to all scanning frames, standardizing the data. Subsequently, overlapping space–time windows are generated by applying a sliding window with a length equal to the scanning period and a width equal to the channel width on each scanning frame. For each space–time window, multi-channel time-frequency feature matrices are obtained using the S-transform. These matrices are then used for training the proposed STNet, and the resultant training weights are saved.

During the testing phase, the training-set standardization parameters are used for standardizing the test-set data, while the weights of the trained model saved at the end of the training phase are utilized for preprocessing the standardized test-set data. Then, the intrusion detection and classification of the space–time windows is performed. Finally, the candidate boxes for the intrusion events are processed using the NMSA, and the classification results are obtained.

### 2.2. Time-Frequency Analysis and the Stockwell Transform

Time-frequency analysis is central to the processing of non-stationary signals. In this analysis, the analyzed signal is represented as a two-dimensional function of time and frequency, capturing the possible temporal change in the signal’s frequency. DAS vibration signals are typically non-stationary; therefore, time-frequency analysis methods can in principle be used for effectively extracting specific information characterizing these vibration signals. The S-transform is a time-frequency analysis method [23] that is often used for analyzing medical signals [24,25], motor fault signals [26], power quality interference events [27], and seismic signal simulations [28]. The S-transform has a good time-frequency resolution and positioning ability with respect to strongly non-stationary signals with abruptly changing characteristics. Therefore, this study used the S-transform for time-frequency analysis.

The S-transform is a Fourier-related transform that is used for determining the sinusoidal frequency and phase content of the local sections of a signal as it changes with time. In practice, the S-transform matrix is computed by dividing the analyzed signal into shorter equal-length segments using a windowing function, called the Gaussian window function; then, the Fourier transform is computed separately on all the short segments. Mathematically, this is expressed as follows:

(1) $$\begin{array}{cc} S(\tau ,f) & =\int_{-\infty }^{\infty }h\left(t\right)\omega \left(t-\tau \right)e^{-i2\pi ft}\;dt \end{array}$$

where S(τ,f) is the S-domain time-frequency spectrum matrix obtained by calculating the vibration signal h(t) of a channel, τ is the translation factor for controlling the position of the Gaussian window on the time axis *t*, *f* is the frequency, *i* is the imaginary unit, and ω(t−τ) is a Gaussian window function expressed as

(2) $$\begin{array}{cc} \omega (t-\tau ) & =\frac{\left|f\right|}{\sqrt{2\pi }}e^{-\frac{{\left(t-\tau \right)}^{2}f^{2}}{2}} \end{array}$$

The superiority of the S-transform is evident from Equation (1), which highlights that the S-transform employs a frequency-dependent Gaussian window function instead of using a fixed window (as is the case for the Fourier transform). The width of the Gaussian window function is proportional to the reciprocal of the frequency, enabling narrow windows at high frequencies and wide windows at low frequencies. This improves the time resolution at high frequencies and improves the frequency resolution at low frequencies of the signal.

**Figure 2:**
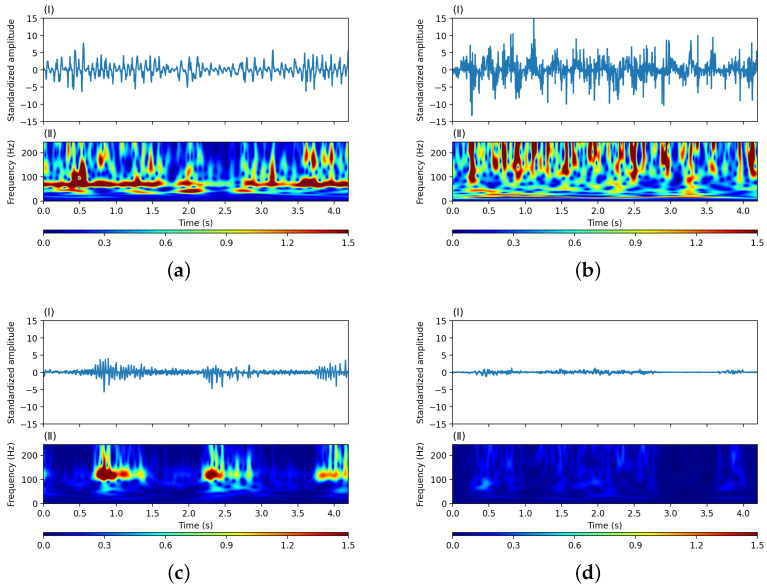
Waveform and time-frequency diagrams of three types of typical intrusion event signals and a clean background signal: (**a**) signal corresponding to climbing a fence; (**b**) signal corresponding to shaking a barbed wire; (**c**) signal corresponding to breaking a wall; (**d**) clean background (I) original time-domain waveform, (II) time-frequency representation processed by the S-transform.

Fourier-related transforms generally handle signal lengths that are exponential times 2; thus, we used sampling points $t\in \left\{256,\;512,\;1024,\;2048\right\}$ in our experiments. Three typical intrusion event signals were collected (climbing a fence, shaking a barbed wire, and breaking a wall). Additionally, a clean background signal was collected in a realistic high-speed railway environment. The results of converting the time-domain signals of a single channel into the time-frequency representation using the S-transform are shown in Figure 2. The signal sampling rate was 488 Hz using our DAS system, and the total length of the signal was equivalent to 2048 sampling points, corresponding to approximately 4.19 s.

### 2.3. Data Preprocessing

**Figure 3:**
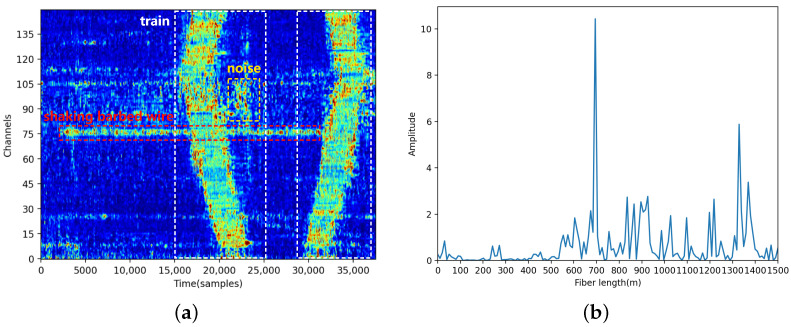
(**a**) Intrusion and interference signals (fs = 488 Hz, channels = 150). (**b**) A corresponding OTDR trace recorded by the DAS system (t = 17,500 samples).

**Figure 4:**
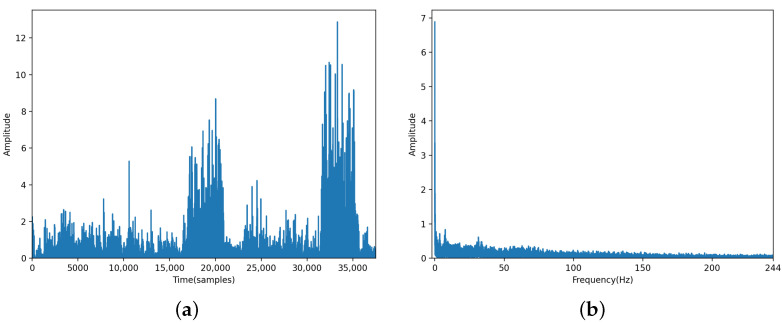
Time domain (**a**) and frequency domain (**b**) of the vibration signal acquired at the 75th channel.

Over time, the DAS system collects signals that cover a certain number of channels. The vibration signals of a typical intrusion event captured by the DAS system are shown in Figure 3a, in which the horizontal and vertical axes represent the time and channel number, respectively. The red dotted box indicates the signal corresponding to shaking a barbed wire, whereas the white and yellow dotted boxes indicate the signals corresponding to a train and unknown interference noise, respectively. Figure 3b shows a corresponding OTDR trace (t = 17,500 samples). Furthermore, Figure 4a,b show the time-domain and frequency-domain data of the vibration signal acquired at the 75th channel, respectively. The sample shown in Figure 3a has a total of 150 channels, and collected signal data of all these channels constitute the visualization result shown in Figure 3a.

In this study, the signals collected by the DAS system were processed at certain time intervals, which were defined as the scanning periods. The data that were acquired during a scanning period covering the entire channel range constituted a scanning frame. A scanning frame captured the signal changes over the entire range of monitoring channels in a certain scanning period.

**Figure 5:**
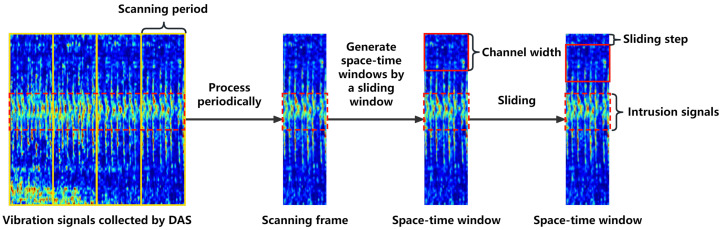
Generation of overlapping space–time windows.

In a scanning frame, the vibration signals of intrusion events might appear in multiple channels. To better locate the range of channels in which intrusions were likely to occur, a space–time window was used, with the same length as the scanning period; the window was slid in the channel direction of the scanning frame. The width of the space–time window was defined as the channel width and was typically composed of multiple channels. The sliding step of the window was 1/5 of the channel width. Therefore, when the window slid on the scanning frame, many overlapping space–time windows were generated. For each space–time window generated by sliding the scanning frame, the time-frequency features of a single channel were extracted using the S-transform and then combined into a time-frequency feature matrix of multiple channels. Figure 5 shows the generation process of such overlapping space–time windows.

Subsequently, z-score standardization was performed for all scanning frames. Mathematically, this standardization can be expressed as

(3) $$z=\frac{x-\mu }{\sigma }$$

where *x* denotes the original scanning frame, μ is the mean value of *x*, σ is the standard deviation of *x*, and *z* is the converted z-score of *x*. μ and σ are expressed as follows:

(4) $$\begin{array}{cc} \mu & =\frac{1}{n}\sum_{i=0}^{n-1}x_{i}\\ \sigma & =\frac{1}{n}\sum_{i=0}^{n-1}{\left(x_{i}-\mu \right)}^{2} \end{array}$$

where *n* is the total number of scanning frames.

### 2.4. STNet

The signal in the DAS system propagates within a certain channel range, while receiving coherent interference and a large amount of noise from moving objects. Distinguishing interference generated by passing vehicles or other noise sources from intrusion threat signals increases the difficulty of the task. The signals of different behaviors received by DAS have different patterns in both the time-frequency domain and space domain. This observation inspires us to combine multi-channel time-frequency features to identify intrusions and reduce the impact of noise.

As a feed-forward neural network, CNN can effectively capture features in two main steps: (1) feature extraction and (2) classification. These steps are characterized by different functional organizations. The part of a CNN responsible for the feature extraction typically contains multiple feature layers, with each feature layer composed of convolutional layers and pooling layers. For classification, a classifier is typically used, such as a fully connected network or an SVM, which is presented with the output of the last feature layer for complete classification.

**Figure 6:**
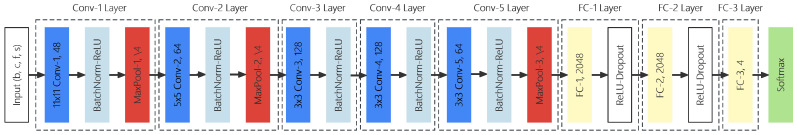
Structure of the proposed STNet for intrusion detection and classification.

The structure and parameters of the Stockwell transform-based network (STNet) proposed in this paper are slightly different according to the scanning period and channel width. An example is shown in Figure 6. More detailed parameters of the proposed STNet are listed in Table 1.

The proposed STNet is composed of an input layer, five convolutional layers, and three fully connected layers. Each convolutional layer is connected to an activation function, namely, ReLU, and a max-pooling layer (except for the third and fourth layers). In addition, the BatchNorm layer is used to accelerate the network convergence, and dropout is used to mitigate overfitting. The S-transform is performed on the signal of each channel in the space–time window, and then the single-channel features are stacked into a multi-channel time-frequency feature matrix according to the channel dimension. The input layer inputs this kind of feature matrix with dimensions b×c×f×s, where *b* is the mini-batch size, *c* is the channel width of the window, *f* is the frequency range of the signal, and *s* is the number of sampled points. Thus, STNet not only captures the time-frequency depth characteristics of the signal, but also captures the spatial relations of the signal from multiple channels.

### 2.5. Non-Maximum Suppression

**Figure 7:**
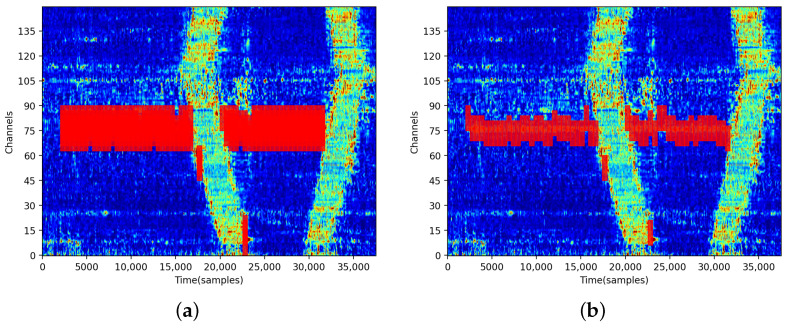
Intrusion detection results before (**a**) and after (**b**) NMS, where the red boxes are windows with intrusions detected by the proposed STNet.

For the overlapping space–time windows generated by sliding in a scanning frame, many candidate intrusion event-containing boxes are typically proposed by the method. As shown in Figure 7a, the candidate boxes typically overlap, which makes generating final predictions difficult. To resolve this issue, the proposed method uses the NMSA for removing redundant candidate boxes.

The algorithm accepts as input set *B* containing candidate boxes, with corresponding confidence levels in set *C*. Then, empty set *R* is initialized as a container for the output. After determining the maximum confidence *m* from *C* and the candidate box *M* corresponding to *m* from *B* each time, the corresponding box is deleted from the list and added to output set *R*. Simultaneously, candidate boxes whose intersection over union (IoU) with *M* is above some threshold $N_{t}$ are deleted as well. This process is repeated until *B* becomes an empty set. The procedure is shown in the following flowchart, Algorithm 1.

In the target-detection literature, the threshold value of $N_{t}$ is typically in the 0.3–0.5 range [29]. Considering that intrusion events should be localized with the maximal possible precision, in the present study, the threshold value was set to 0. The results obtained after running the NMSA are shown in Figure 7b.
**Algorithm 1** NMSA for intrusion detection**Input:** $B=\{b_{1},...,b_{n}\}$, $C=\{c_{1},...,c_{n}\}$, $N_{t}$  1: R←⌀  2: **repeat**  3:    m←max(C)  4:    $M\leftarrow b_{m}$  5:    R←R⋃M  6:    B←B−M  7:    **for** $b_{i}$ in *B* **do**  8:        **if** $IoU\left(M,b_{i}\right)\geq N_{t}$ **then**  9:           $B\leftarrow B-b_{i}$ 10:           $C\leftarrow C-c_{i}$ 11:        **end if** 12:    **end for** 13: **until** B=⌀ **Output:** *R*, *C*


## 3. Experimental Procedure

### 3.1. Principle of the DAS System

**Figure 8:**
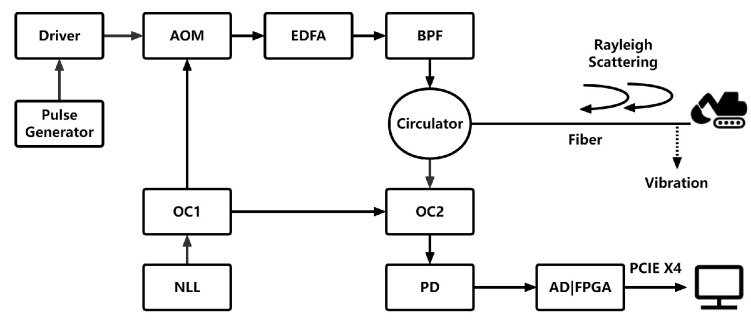
Structure of the DAS system.

The DAS system is shown schematically in Figure 8, which applies phase-sensitive optical time-domain reflectometry (Φ-OTDR). Φ-OTDR launches periodic pulses to detect the disturbance of the fiber continuously. Thus, vibrations generated by intrusions or the running trains will propagate to the fiber and can be detected by Φ-OTDR in the DAS system.

The experimental system used a narrow linewidth laser (NLL) (wavelength, 1550 nm; linewidth, 3 kHz; maximum laser power, 13 dBm) as the laser source. The continuous light wave emitted by the laser source was divided into two beams by optical coupler 1 (OC1). The first beam contained 98% of the original beam. It was modulated into a pulse by an acoustic–optic modulator (AOM), yielding a frequency shift of 80 MHz. The pulse generator generated a TTL pulse sequence with a period of 0.512 ms and a width of 100 ns to drive the AOM. Simultaneously, to reduce the optical power loss in long-distance transmission, the pulsed light beam was amplified by an erbium-doped fiber amplifier (EDFA), and spontaneous emission noise generated by the amplifier was suppressed using a bandpass filter (BPF). The pulsed light beam was emitted from a circulator into a buried single-mode optical fiber where small variations in the refractive index lead to the scattering of light called the Rayleigh backscattered light (RBL) along the fiber. The second beam accounted for 2% of the original beam. It was mixed with the RBL signal that returned from the circulator to the original point of the optical fiber through the 3dB optical coupler 2 (OC2). The mixed signal was detected by a photodetector (PD) (bandwidth, 200 MHz) and was sampled by a 12-bit 250 MSps AD chip controlled by the FPGA. The frequency, amplitude, and phase of the mixed signal were obtained in a field-programmable gate array (FPGA by Xilinx Artix-7) through fast Fourier transform. The phase of the mixed signal was decimated 25 times and averaged by four successive interrogate periods, taking the requirements for intrusion detection and the calculation ability of the upper computer into account. As a result, the final spatial resolution of the DAS system was 10.2 m, the time interval between adjacent channels was 100 ns, and the final sampling frequency (fs) was 488 Hz. The channels in the DAS system can be understood as multiple sensors (i.e., multiple continuous channels) along the optical fiber, and the arrangement of channels is determined by the arrangement of buried optical fibers. The distance between each channel is the spatial resolution of DAS, and the data received by each channel are different. When the vibration generated by the intrusion occurred between two unstrained locations, the phase difference between the two regions varied linearly with the vibration. The phase difference was calculated in the FPGA and transmitted to the upper personal computer (PC) through the peripheral component interconnect express (PCIE) X4 port.

The above parameters were determined after multiple on-site experiments, taking into account factors such as the DAS system’s storage capacity, signal collection, and calculation pressure.

### 3.2. Experimental Setup

Field experiments based on the DAS system presented in Figure 8 were conducted at an actual high-speed railway site [10]. To record experimental data, the DAS system was deployed at a railway station with multiple tracks passing in parallel. During the experiment, trains passed through the high-speed railway station. A typical high-speed rail environment is characterized by strong noise interference, owing to passing trains, heavy trucks, and other noise sources. The spatial resolution of the DAS system was 10.2 m, with a maximum sensing range of up to 40 km, and the sampling frequency of the signal was 488 Hz. Considering the existence of a large amount of invalid data within the actual monitoring range, the dataset only included 70–150 channels (corresponding to approximately 0.7–1.5 km) containing intrusion-related events. The optical fiber used in the system was affixed to a separation wall along the high-speed railway, which runs parallel to the tracks, and a barbed wire was placed atop the separation wall [10]. The DAS vibration signal data for three different intrusion events were recorded during the experiment: (1) climbing a fence, (2) shaking a barbed wire, and (3) breaking a wall. Several background signals were captured simultaneously. In detail:

(1) Climbing the fence: A person climbed over the fence on the separation wall. On average, this action lasted for approximately 24 s.
(2) Shaking the barbed wire: A person shook the barbed wire atop the isolation wall. On average, this action lasted for approximately 40 s.
(3) Breaking the wall: A person used a hammer to hit the isolation wall approximately once every second. On average, this action lasted for approximately 35 s.
(4) Background: Background signals were captured in a relatively sunny environment near the sensing fiber and consisted of noise generated by passing trains and heavy trucks, as well as by other noise sources.

Three types of intrusion events that often occur in high-speed rail environments were simulated to ensure that manually marked intrusion signals were associated with the above three types.

### 3.3. Dataset and Network Training

In total, 152 samples were collected in the experiment. Table 2 lists the detailed information about the dataset.

In total, 81 samples were used as the training set and 71 samples as the testing set. Table 3 shows the number of windows of the training and testing sets of different channel width with the scanning period set to 512, the approximate proportions of which are 55% and 45%, respectively.

**Figure 9:**
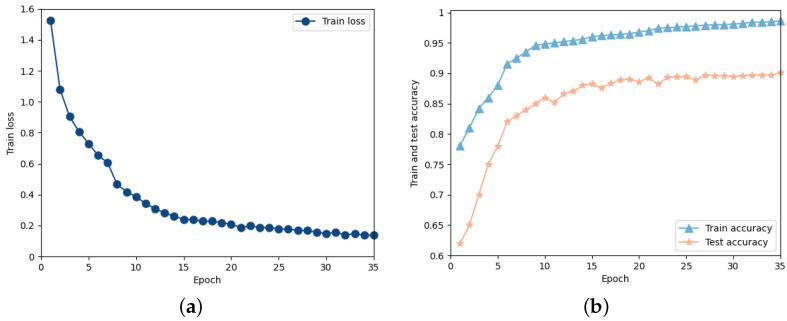
The train loss (**a**) and train/test accuracy (**b**) curves for STNet.

We used this dataset to train the proposed STNet. The neural network model was implemented using the PyTorch framework and run on an NVIDIA GeForce GTX 1660 Ti with 6 GB of onboard memory. The batch size, dropout value, and learning rate were 64, 0.5, and 0.0001, respectively. The training process was run for 35 epochs and the last epoch was used to determine the model parameters for initializing the model before testing. Figure 9 shows the train loss and train/test accuracy curves of STNet.

The proposed STNet evaluated each space–time window represented by the time-frequency feature matrix after the S-transform and output four types of labels, corresponding to climbing a fence, shaking a barbed wire, breaking a wall, and background (including noise sources), respectively:

(5) $$classification\left(w\right)=\left\{\begin{array}{ccc} 1 & \; & climbing\;fence\\ 2 & \; & shaking\;barbed\;wire\\ 3 & \; & breaking\;wall\\ 0 & \; & background \end{array}\right.$$

where *w* is the space–time window to be detected.

The detected windows labeled with 1, 2, or 3 indicated intrusions:

(6) $$detection\left(w\right)=\left\{\begin{array}{ccc} intrusion & \; & label_{w}\;=\;1,\;2\;or\;3\\ non\text{-}intrusion & \; & label_{w}\;=\;0 \end{array}\right.$$

The proposed model used the Adam optimizer and cross-entropy as loss functions:

(7) $$\begin{array}{c} Loss=\frac{1}{N}\sum_{i=1}^{N}\sum_{j=1}^{K}-y_{ij}\log\hat{y_{ij}}-\left(1-y_{ij}\right)\log\left(1-\hat{y_{ij}}\right) \end{array}$$

where Loss represents the loss function, *N* is the overall number of space–time windows, and *K* is the number of classes. $y_{ij}$ is the true label of space–time window *i* belonging to class *j*. $\log\hat{y_{ij}}$ represents the probability that window *i* belongs to class *j*.

### 3.4. Evaluation Metrics

During the testing phase, we first calculated the numbers of true positives (TPs), false negatives (FNs), and false positives (FPs) for each scanning frame processed using the NMSA, where

TP corresponded to the situation in which the candidate box correctly overlapped the ground-truth box. (In the case of multiple overlapping candidate boxes, only one TP was counted.)
FN corresponded to the case in which a ground-truth box existed in the scanning frame, but none of the candidate boxes hit it.
FP corresponded to the case in which the candidate box did not intersect with any ground-truth box in the scanning frame.

The TP, FN, and FP numbers were summarized over all of the scanning frames, and the performance of the proposed method was quantified using the following metrics:

(1) Intrusion detection rate (IDR): the fraction of detected intrusion events, equivalent to recall:
  (8) $$IDR=\frac{TP}{TP+FN}=recall$$
(2) False alarm rate (FAR): the ratio of the number of false alarms to the number of all triggered alarms, corresponding to 1-precision:
  (9) $$FAR=1-\frac{TP}{TP+FP}=1-precision$$
(3) F_1_ score: the harmonic mean of the IDR and 1 − FAR, for measuring the overall performance of a method:
  (10) $$F_{1}=\frac{2\times precision\times recall}{precision+recall}=\frac{2\times (1-FAR)\times IDR}{(1-FAR)+IDR}$$

## 4. Experimental Results

### 4.1. Effects of the Scanning Period and Channel Width

This study compared the effects of different channel widths and scanning periods on the intrusion detection performance for channel widths in the 10–30 range and scanning periods in the 256–2048 range. Table 4 lists the intrusion classification and detection results varying with the channel width. Table 5 lists the intrusion classification and detection results varying with the scanning period.

As shown in Table 4, for a fixed scanning period, the IDR values for various channel widths were similar, with an average value of approximately 96.0%. A clear dependence on the channel width was observed in the case of the FAR metric. Increasing the window channel width improved the FAR, thus improving the overall classification and detection accuracy of the system. Clearly, wider channels implied more spatial information, which improved the overall performance.

From Table 5, we see that, for a fixed channel width of the space–time window, the system’s performance did not linearly depend on the scanning period. The overall performance of the system was the best when the scanning period was 512 sampling points. In addition, as the scanning period increased, the FAR gradually increased, implying that for long-duration signals, the time-frequency feature matrices extracted by the S-transform were not clearly distinguishable by the proposed STNet for detection and classification.

**Figure 10:**
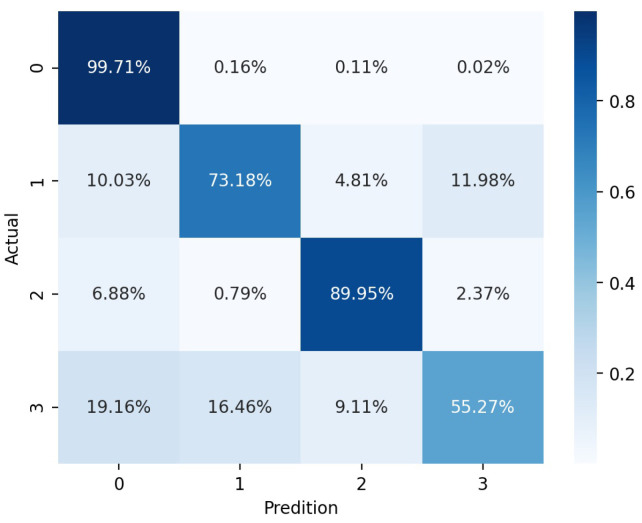
Confusion matrix for intrusion classification (channel width = 15, scanning period = 512); 0: background; 1: climbing a fence; 2: shaking a barbed wire; 3: breaking a wall.

Regarding the intrusion classification, the overall recognition results for breaking a wall were not as good as those for the other two categories. From Figure 10, the classification result for shaking a barbed wire was the best, at 89.95%, whereas the result for breaking a wall was the worst, implying that it can be easily confused with fence-climbing and background signals. The intrusion signal owing to the wall-breaking event featured a certain interval, as is evident from Figure 2c. After the intrusion action, which generally lasted for approximately 0.75 s, there was an approximately 1 s long window during which no signals were detected. Therefore, when dividing the data into scanning frames, some time-frequency feature matrices extracted by the S-transform failing to capture intrusion-related signals can likely be obtained with a certain probability.

### 4.2. Comparison of Time-Frequency Analysis Methods

**Figure 11:**
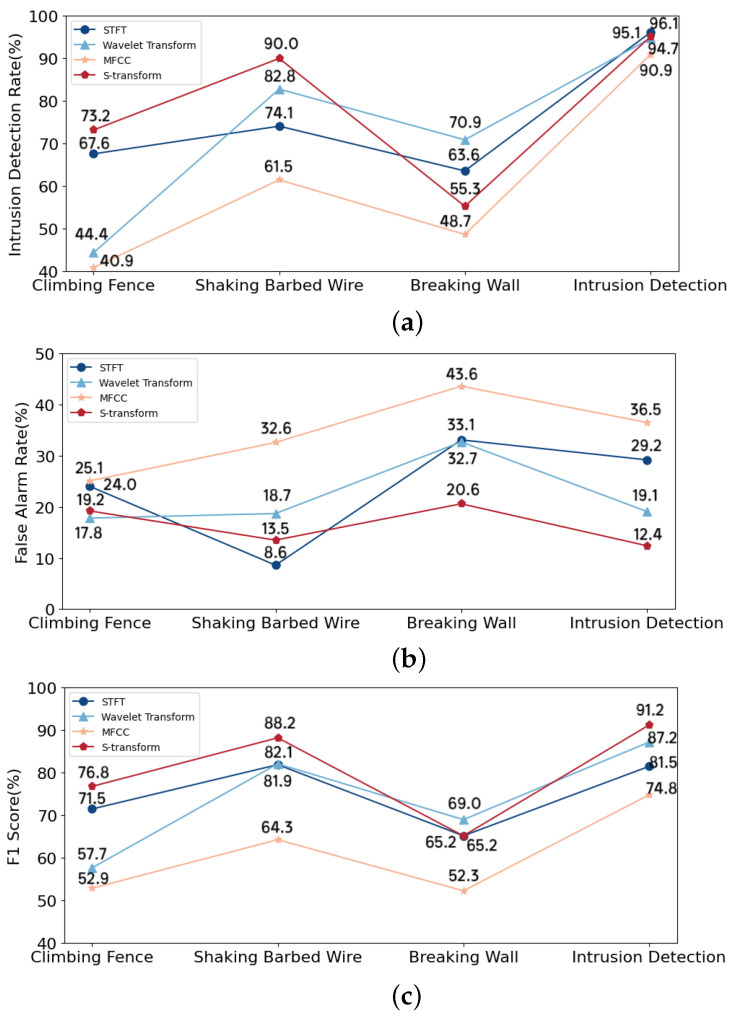
Intrusion detection and classification results for different time-frequency analysis methods: IDR (**a**), FAR (**b**), and F_1_ score-based (**c**) methods. For (**a**,**c**), the higher the value, the stronger the effect. For (**b**), the lower the value, the stronger the effect.

This study compared the S-transform with three other commonly used time-frequency analysis methods, namely, the STFT, the wavelet transform, and the method utilizing MFCCs. The results are presented in Figure 11. The window function in the STFT was the Hamming window, the number of FFT bins was 128, the time length of each frame was 128 sampling points, and the overlap rate between the frames was 50%. The wavelet transform used Cgau1 as the wavelet basis function, and the wavelet scale was 246. To calculate the MFCCs of vibration signals, the frame length was 25 ms and the overlap was 10 ms. The time-frequency matrices obtained using the three methods were presented to the proposed STNet.

From Figure 11, it is evident that the overall performance of the S-transform was better than those of the other three methods, suggesting that the S-transform effectively captured the time-frequency characteristics of intrusion signals and was insensitive to noise. As a frequently adopted approach in the field of speech recognition, MFCCs can also reflect the signals’ time-frequency characteristics to a certain extent. However, the results generated using MFCCs were not as good as those generated using the other three time-frequency analysis methods.

### 4.3. Comparison of Model Performances

To verify the performance of the proposed model, we compared the proposed STNet with other common algorithms and models. Collected in an actual high-speed railway environment, our dataset contained certain environmental noise and interference signals. Reducing the FAR of the interference noise while ensuring a high intrusion detection rate is challenging. The algorithms and models tested in this study included commonly used deep-learning networks, such as AlexNet [30], VGG16 [31], Inception-v3 [32], ResNet [33], and DenseNet [34]. In our comparison, these models directly used the presented space–time windows as the input to them. Based on the DAS-related literature, we compared the proposed STNet with a ConvLSTM-based model for detecting intrusions in high-speed rail environments [10]. We also compared our proposed model with the pattern-recognition model for detection of vibrational events based on the STFT and CNN [19], with the event-recognition method for optical fiber sensors based on MFCCs and AlexNet [20], and with the attention-based network called ATCN-BiLSTM for Φ-OTDR event classification [21]. The results are shown in Table 6.

The results in Table 6 demonstrate that the performance of the proposed STNet was comparable to that of several other models in terms of the IDR, falling only 2.6% below that of the top-performing model. However, in terms of the FAR, our model outperformed the second-best model by nearly 11%, which demonstrates that the proposed model can significantly reduce misclassification owing to the interference noise. Furthermore, on the intrusion classification task, our model outperformed the other models overall, except for the scenario of breaking a wall, in which the proposed model was 28.7% worse than the best model in terms of the IDR. The models [10,30,31,32,33,34] detect and classify signals by extracting spatiotemporal features. However, distinguishing intrusion signals from noise can be challenging, owing to the similarity of their spatiotemporal features. In our experiment, this was particularly true for strong background noise of unknown origin. Consequently, the average FAR on the intrusion detection task was 38.4%. Similarly, the ATCN-BiLSTM [21] model focused only on the signal specificity in the time domain and was unable to differentiate interference signals from intrusion signals, resulting in a low IDR and high FAR of 17.4% and 63.6%, respectively. The model in [19] considered the time-frequency characteristics of the detected signal; however, the performance of that method was not as good as that of our proposed method, with the IDR and FAR lower by approximately 27.7% and 17.8%, respectively, compared with the proposed STNet on the intrusion detection task. The model in [20] overlay the MFCC features and spatiotemporal data of the signals. Compared with the models in [30,31,32,33] that used only spatiotemporal information, the FAR on the intrusion detection task was lower, but the problems identified in this study remained inadequately addressed.

## 5. Conclusions and Discussion

This paper proposed a general method based on the S-transform and STNet for the detection and classification of DAS intrusion-related vibration signals in highly noisy environments.

To detect and classify the vibration signals collected by the DAS system, we first decomposed the signal-containing samples into spatiotemporal windows using sliding windows. We then used the S-transform to extract the time-frequency features from these windows and presented them to the proposed STNet. Finally, we used the NMSA to post-process the detection results. In the experiments conducted in a real high-speed railway environment with high levels of interference noise, we artificially simulated three types of intrusion behavior: (1) climbing a fence, (2) shaking a barbed wire, and (3) breaking a wall. Our proposed method achieved the maximal IDR of 96.9%, while the FAR was only 4.3%. Our method outperformed other existing methods by significantly reducing the FAR while maintaining high detection accuracy. These results demonstrated the effectiveness of the proposed approach.

Considering the trade-off between the signal collection and calculation pressure of the DAS system, this paper set the sampling rate of the DAS system to 488 Hz, which may result in some signals (such as shaking the cage) having frequencies higher than the Nyquist frequency. Therefore, in future work, it is necessary to improve the computational performance and sampling frequency of DAS systems to prevent signal aliasing issues. In addition, owing to the large amount of signal data generated by a typical DAS system and the uncertainty associated with intrusions, manual annotation inevitably causes errors and is time-consuming and labor-intensive. Therefore, weak supervised learning will be considered in future studies.

## Figures and Tables

**Table 1:** Parameters of STNet with input of scanning period of 512 and channel width of 15.

Layer	Kernel Size	Stride	Padding	Output Shape
Conv-1	11	1	1	(48, 235, 502)
MaxPool-1	4	3	0	(48, 78, 167)
Conv-2	5	1	2	(64, 78, 167)
MaxPool-2	4	3	0	(64, 25, 55)
Conv-3	3	1	1	(128, 25, 55)
Conv-4	3	1	1	(128, 25, 55)
Conv-5	3	1	1	(64, 25, 55)
MaxPool-3	4	3	0	(64, 8, 18)
FC-1	-	-	-	(2048)
FC-2	-	-	-	(2048)
FC-3	-	-	-	(4)

**Table 2:** Details of the dataset collected in our experiment (fs = 488 Hz).

Information	Climbing Fence	Shaking Barbed Wire	Breaking Wall
Number of samples	53	52	47
Minimum number of sampling points	7500	10,000	4500
Maximum number of sampling points	40,500	40,500	37,000
Average number of samping points	15,755	23,337	20,798
Minimum duration of intrusion	2683	4820	4316
Maximum duration of intrusion	28,269	40,439	31,598
Average duration of intrusion	11,503	19,386	16,849
Minimum number of channels	70	70	70
Maximum number of channels	150	150	150
Average number of channels	127	125	122
Minimum number of invaded channels	8	4	7
Maximum number of invaded channels	27	15	19
Average number of invaded channels	17	7	13

**Table 3:** Number of windows generated with different channel widths (fs = 488 Hz, scanning period = 512).

Channel Width	Training Set	Testing Set
10	199,440	159,295
15	128,246	102,428
20	92,855	73,976
25	70,131	55,819
30	56,131	44,680

**Table 4:** Intrusion detection and classification results for different channel widths (scanning period = 512). The best results are in bold font. IDR: intrusion detection rate. FAR: false alarm rate. F_1_:F_1_ score.

Channel Width	Intrusion Classification									Intrusion Detection		
	Climbing Fence			Shaking Barbed Wire			Breaking Wall			IDR	FAR	F_1_
	IDR	FAR	F_1_	IDR	FAR	F_1_	IDR	FAR	F_1_			
10	68.5%	16.2%	75.4%	80.4%	11.0%	84.5%	**77.0%**	27.2%	74.8%	**96.9%**	23.9%	85.2%
15	**73.2%**	19.2%	76.8%	**90.0%**	13.5%	**88.2%**	55.3%	20.6%	65.2%	95.1%	12.4%	91.2%
20	66.6%	13.4%	75.3%	83.1%	14.5%	84.3%	71.2%	23.8%	73.6%	96.0%	15.3%	90.0%
25	71.9%	14.7%	78.1%	73.4%	**9.9%**	80.9%	74.2%	22.7%	75.7%	95.4%	12.2%	91.4%
30	71.4%	**8.8%**	**80.1%**	86.0%	13.0%	86.5%	75.4%	**19.1%**	**78.1%**	96.6%	**11.6%**	**92.3%**

**Table 5:** Intrusion detection and classification results for different scanning periods (channel width = 15). The best results are in bold font.

Scanning Period	Intrusion Classification									Intrusion Detection		
	Climbing Fence			Shaking Barbed Wire			Breaking Wall			IDR	FAR	F_1_
	IDR	FAR	F_1_	IDR	FAR	F_1_	IDR	FAR	F_1_			
256	58.5%	18.1%	68.3%	77.1%	16.8%	80.0%	68.7%	31.3%	68.7%	76.9%	**4.3%**	85.3%
512	**73.2%**	19.2%	**76.8%**	**90.0%**	**13.5%**	**88.2%**	55.3%	**20.6%**	65.2%	95.1%	12.4%	**91.2%**
1024	68.0%	**15.5%**	75.6%	72.9%	17.6%	77.4%	**71.0%**	26.5%	**72.2%**	94.7%	21.9%	85.6%
2048	60.2%	26.0%	66.4%	71.6%	32.6%	69.5%	48.9%	35.2%	54.4%	**96.6%**	54.1%	62.3%

**Table 6:** Intrusion detection and classification results for the proposed STNet and other models (scanning period = 512, channel width = 15). The best results are in bold font.

Model		Intrusion Classification									Intrusion Detection		
		Climbing Fence			Shaking Barbed Wire			Breaking Wall			IDR	FAR	F_1_
		IDR	FAR	F_1_	IDR	FAR	F_1_	IDR	FAR	F_1_			
Time	ATCN-BiLSTM [21]	23.5%	55.1%	30.8%	12.8%	74.1%	17.2%	15.9%	61.5%	22.5%	35.8%	23.6%	48.7%
Space– Time	AlexNet [30]	54.6%	35.3%	59.2%	27.5%	18.9%	41.1%	65.3%	49.2%	57.1%	**97.6%**	54.3%	62.3%
	VGG16 [31]	71.7%	34.0%	68.7%	50.2%	15.5%	63.0%	51.5%	40.9%	55.1%	96.8%	47.6%	68.0%
	Inception-v3 [32]	41.1%	21.2%	54.0%	40.7%	17.8%	54.4%	**84.0%**	43.1%	**67.9%**	96.4%	48.3%	67.3%
	ResNet [33]	36.2%	28.0%	48.2%	57.6%	24.3%	65.4%	71.5%	42.2%	63.9%	95.1%	46.2%	68.7%
	DenseNet [34]	62.6%	31.6%	65.4%	45.6%	**10.9%**	60.3%	72.1%	36.3%	67.6%	96.2%	36.0%	76.9%
	ConvLSTM [10]	54.6%	29.0%	61.7%	70.6%	28.6%	71.0%	45.7%	38.5%	52.5%	93.6%	30.4%	80.0%
Time- Frequency	Xu [19]	44.6%	56.8%	43.9%	45.6%	55.5%	45.1%	54.4%	38.2%	57.9%	67.4%	30.2%	68.6%
	Shi [20]	43.8%	52.1%	45.7%	64.3%	74.2%	36.8%	61.7%	45.0%	58.2%	71.9%	37.8%	66.7%
	STNet	**73.2%**	**19.2%**	**76.8%**	**90.0%**	13.5%	**88.2%**	55.3%	**20.6%**	65.2%	95.1%	**12.4%**	**91.2%**

## Data Availability

Due to privacy restrictions, the dataset has not been published yet.
